# Herbal medicine for the treatment of obesity-associated asthma: a comprehensive review

**DOI:** 10.3389/fphar.2023.1186060

**Published:** 2023-05-11

**Authors:** Aparoop Das, Manash Pratim Pathak, Kalyani Pathak, Riya Saikia, Urvashee Gogoi

**Affiliations:** ^1^ Department of Pharmaceutical Sciences, Dibrugarh University, Dibrugarh, Assam, India; ^2^ Faculty of Pharmaceutical Science, Assam Down Town University, Guwahati, Assam, India

**Keywords:** obesity-associated asthma, herbal medicine, bioactive phytoconstituents, adiponectin, macrophage polarization, NLRP3, NOTCH1

## Abstract

Obesity is fast growing as a global pandemic and is associated with numerous comorbidities like cardiovascular disease, hypertension, diabetes, gastroesophageal reflux disease, sleep disorders, nephropathy, neuropathy, as well as asthma**.** Studies stated that obese asthmatic subjects suffer from an increased risk of asthma, and encounter severe symptoms due to a number of pathophysiology. It is very vital to understand the copious relationship between obesity and asthma, however, a clear and pinpoint pathogenesis underlying the association between obesity and asthma is scarce. There is a plethora of obesity-asthma etiologies reported viz., increased circulating pro-inflammatory adipokines like leptin, resistin, and decreased anti-inflammatory adipokines like adiponectin, depletion of ROS controller Nrf2/HO-1 axis, nucleotide-binding domain, leucine-rich-containing family, pyrin domain-containing-3 (NLRP3) associated macrophage polarization, hypertrophy of WAT, activation of Notch signaling pathway, and dysregulated melanocortin pathway reported, however, there is a very limited number of reports that interrelates these pathophysiologies. Due to the underlying complex pathophysiologies exaggerated by obese conditions, obese asthmatics respond poorly to anti-asthmatic drugs. The poor response towards anti-asthmatic drugs may be due to the anti-asthmatics approach only that ignores the anti-obesity target. So, aiming only at the conventional anti-asthmatic targets in obese-asthmatics may prove to be futile until and unless treatment is directed towards ameliorating obesity pathogenesis for a holistic approach towards amelioration of obesity-associated asthma. Herbal medicines for obesity as well as obesity-associated comorbidities are fast becoming safer and more effective alternatives to conventional drugs due to their multitargeted approach with fewer adverse effects. Although, herbal medicines are widely used for obesity-associated comorbidities, however, a limited number of herbal medicines have been scientifically validated and reported against obesity-associated asthma. Notable among them are quercetin, curcumin, geraniol, resveratrol, *β*-Caryophyllene, celastrol, tomatidine to name a few. In view of this, there is a dire need for a comprehensive review that may summarize the role of bioactive phytoconstituents from different sources like plants, marine as well as essential oils in terms of their therapeutic mechanisms. So, this review aims to critically discuss the therapeutic role of herbal medicine in the form of bioactive phytoconstituents against obesity-associated asthma available in the scientific literature to date.

## 1 Introduction

**FIGURE 1:**
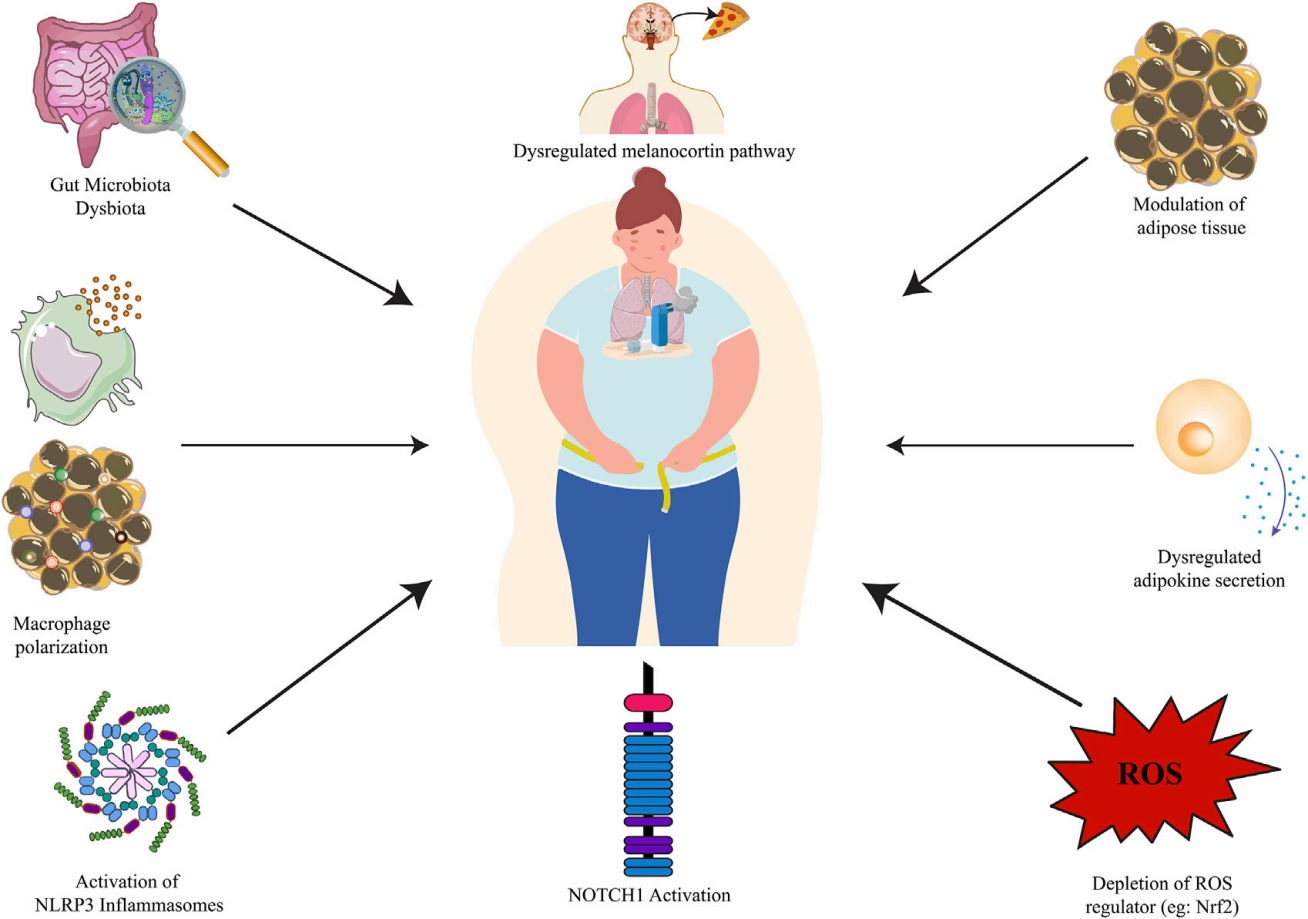
Obesity-asthma pathophysiology. NLRP3: nucleotide-binding domain, leucine-rich-containing family, pyrin domain-containing-3; NOTCH1: Neurogenic locus notch homolog protein 1.

Obesity affects more than one billion people worldwide, including 650 million adults, 340 million adolescents, and 39 million children, and the number is alarmingly rising (World Health Organization, 2022). Hippocrates, the Greek Physician in the classical Greek era quoted that “Corpulence is not only a disease itself but the harbinger of others,” which holds true in today’s modern world. Obesity is associated with numerous comorbidities like cardiovascular disease, hypertension, diabetes, gastroesophageal reflux disease, sleep disorders, nephropathy, neuropathy, as well as asthma. As per the latest World Health Organization statistics, ischemic heart disease (IHC) tops the leading causes of death globally, however, reports stated that obesity is one of the prominent causes of IHC (World Health Organization, 2021). Studies stated that obese asthmatic subjects suffer from an increased risk of asthma, and encounter severe symptoms that result in the excessive release of white adipose-derived pro-inflammatory adipokines producing severe oxidative stress in the respiratory system (Global Initiative for Asthma, 2020; Pathak et al., 2021). Obesity and asthma have both increased in the United States over the last several decades with nearly 60% of adults with severe asthma being obese. Asthma prevalence is prominent in obese adult women and children. Obesity has a copious effect on the risk of asthma, the higher the body mass index (BMI), the higher the risk of asthma (Peters et al., 2018; Koebnick et al., 2016). There is a plethora of reported obesity-asthma pathophysiologies, namely, modulation of adipose tissue (Lumeng and Saltiel, 2011), increased circulating pro-inflammatory adipokines like leptin, and resistin, and decreased anti-inflammatory adipokines like adiponectin (Fu et al., 2017; Salvator et al., 2021; Palma et al., 2022), depletion of ROS controller Nrf2/HO-1 axis (Yuan et al., 2018; Pathak et al., 2021), NLRP3 associated macrophage polarization (Russo and Lumeng, 2018), activation of the Notch signaling pathway (Zeng et al., 2019), downregulation of Ucp1 in BAT (Cannon and Nedergaard, 2004) followed by downregulated AMPKα (Yamauchi et al., 2003) and melanocortin pathway (Raap et al., 2003) are reported (Figure 1). Asthma is worsened in obese patients and they become less responsive to conventional anti-asthmatic drugs. Inhaled corticosteroids (ICTs) are the common and most-effective drugs that provide fast relief by acting on the airways. Although effective in non-obese asthmatics, ICT does not provide relief to obese asthmatics, and in fact, increased ICT doses lead to poorer asthma control in the absence of eosinophilic inflammation in the sputum or presence of bronchial neutrophilia (Peerboom et al., 2020). Humankind is bestowed with the greatest gift of nature in the form of herbal medicine which comes with a multi-targeted pathway and fewer side effects. There has been an increase in scientific studies evaluating the value of compounds acquired from herbal medicine, which has traditionally been used in folk medicine. Plant-based compounds such as flavonoids, alkaloids, and terpenoids have been reported to show promising *in-vitro* and *in-vivo* anti-obesity effects through a repertoire of mechanisms, namely, appetite suppression, triglyceride reduction, metabolic rate increase, pancreatic lipase inhibition, etc. Recently piperine, found in Piper species, has been reported to ameliorate obesity via the alteration of the gut microbiota (He et al., 2022a) as well as found to combat pancreatic *β*-Cell apoptosis in obese diabetic mice (He et al., 2022b). Similarly, there have been reports of the therapeutic role of plant extract and their bioactive phytoconstituents in allergic asthma. Solasodine, found in Solanaceae family, is reported to suppress ovalbumin (OVA) induced allergic asthma through modulation of different inflammatory markers (Arora et al., 2022a); piperine is also reported to suppress inflammatory markers and oxidative stress in cigarette smoke-exposed experimental mice (Arora et al., 2022b). Likewise, stamen extract of *Mesua ferrea* L. (Calophyllaceae) from the Calophyllaceae family and root extract of *Clerodendrum serratum* of the genus *Clerodendrum* is reported to ameliorate allergic asthma through the modulation of few asthma-associated cytokines like TNF-α, IL-5, and IL-4. The authors speculated that the ameliorative effect of *M. ferrea* L. may be due to the presence of bioactive phytoconstituents like rhusflavanone, mesuaferrone B, *α*-Amyrin or *β*-Amyrin (Arora et al., 2021) and of *C. serratum* due to compounds like scutellarein, icosahydropicenic acid, oleanolic acid, ursolic acid, and queretaroic acid (Arora et al., 2022a). Similarly, the root extract of *Withania somnifera* demonstrated potential anti-asthmatic properties. The study reported that treatment with root extract of *W. somnifera* downregulated pro-inflammatory cytokines like immunoglobulin E (IgE), IL-4), and TNF-α as well as Histone deacetylase 2 (HDAC2) in the blood and bronchoalveolar lavage fluid (BALF) of the treated animals (Ali et al., 2023). From the above published reports, it is observed that bioactive phytoconstituents like piperine possess both the anti-asthmatic as well as anti-obesity properties separately, however, very few studies are undertaken to study both the properties in the same animal model. Although, herbal medicines are widely used for obesity-associated comorbidities nowadays, however, a very smaller number of herbal medicines in the form of bioactive phytoconstituents have been scientifically validated and reported against obesity-associated asthma. Notable among them are resveratrol, *β*-Caryophyllene, celastrol, and tomatidine. In view of this, there is a dire need for a comprehensive review that may summarize the role of herbal medicines targeting obesity-associated asthma in terms of their therapeutic mechanisms. So, this review aims to critically discuss the therapeutic role of herbal medicine in the form of bioactive phytoconstituents against obesity-associated asthma available in the scientific literature to date.

## 2 Obesity-asthma pathophysiology

### 2.1 Modulation of adipose tissue

The adipose tissue produces cytokines and hormones that regulate metabolism and immunity (Sideleva et al., 2012). Adipose tissue can be divided into 2 major types: white adipose tissue (WAT) and brown adipose tissue (BAT). The largest portion of adipose tissue in most organisms is WAT, which is used for energy storage, whereas BAT plays a major role in thermogenesis in small mammals and neonates (Curat et al., 2004; Fantuzzi, 2005). Lean adipose tissue secretes low levels of proinflammatory cytokines [e.g., IL-6, IL-8, tumor necrosis factor (TNF)-α] and adipokines (e.g., leptin), while producing high levels of the anti-inflammatory adipokine adiponectin. Adipose tissue hypertrophies in obese individuals and becomes infiltrated with macrophages that promote inflammation (Lumeng and Saltiel, 2011) and may exaggerate asthmatic conditions in obese individuals (Shim et al., 2023). Consequently, proinflammatory adipokines are speculated to enhance asthmatic airway inflammation in obesity. A recent report suggests that two significant depots of white adipose tissue, i.e., visceral fat are responsible for the narrowing of bronchial lumina and subcutaneous fat for bronchial wall thickening (Yang et al., 2018).

In metabolic syndrome, mitochondrial dysfunction and defective mitochondrial biogenesis are observed in various organs such as adipose tissue, muscle, liver, and beta islet cells in the pancreas (Kim et al., 2008; Luptak et al., 2019). Factors affecting mitochondrial function include hereditary, environmental, and lifestyle influences. Overeating and high-fat consumption are two of the most common causes of obesity, resulting in nutritional overload, excess electron flux, increased oxidative stress, accumulated partially oxidized substrates, and eventually, damage to the body (Koves et al., 2008). Researchers have found that mitochondrial dysfunction in the airway epithelium was a major contributor to allergic asthma models in mice (Mabalirajan et al., 2008). In a similar model, Aguilera-Aguirre et al. (2009) found that pre-existing mitochondrial dysfunction increased asthma severity. Pattnaik et al. (2016) showed that the levels of asymmetric dimethylarginine (ADMA) in allergically inflamed lungs were extremely high, and IL-4 increased ADMA synthesis via protein arginine methyl transferases and inhibited its degradation by dimethylarginine dimethylaminohydrolase 2. In airway epithelial cells, IL-4 and ADMA worked together to fragment mitochondria, increase mitochondrial ROS release, and reduce mitochondrial mass. As a result of this, obesity and asthma, both of which have high ADMA and IL-4 levels, could potentiate each other by causing mitochondrial dysfunction to become more severe (Bhatraju and Agrawal, 2017). There is increasing evidence that energy metabolism plays a significant role in the development and maintenance of inflammatory conditions such as asthma. Energy metabolism depends heavily on adipose tissue. White adipocytes store energy in the form of triglycerides, and beige/brown adipocytes are involved in heat metabolism (Wu et al., 2012; Harms and Seale, 2013). Several studies have indicated that brown adipose tissue (BAT) can be used as an obesity therapeutic target. BATs are specialized for metabolic thermogenesis mediated by mitochondrial uncoupling protein 1 (UCP1), which uncouples respiration from ATP synthesis (Cannon and Nedergaard, 2004). UCP1 is an inner mitochondrial membrane protein that uncouples oxidative phosphorylation from ATP synthesis through FA/H+ symports (Chouchani et al., 2019). UCP1 expression is mainly driven through β3-adrenoreceptors (β3-AR) stimulation by sympathetically and non-sympathetically produced norepinephrine in thermogenically active adipocytes (Alzaim et al., 2020). Thermogenesis in BAT is due to UCP1, also called thermogenin or SLC25A7 (Dalgaard and Pedersen, 2001). Brown adipose tissue-induced thermogenesis is reported to activate the AMPK pathway which is responsible for the amelioration of asthma in obese subjects (Liu et al., 2023). In cold environments and during the postpartum period, UCP1-mediated thermogenesis is critical for thermoregulation (Tsubota et al., 2019) as well as in regulating adiposity. In both rodents and humans, the activation of UCP1-mediated thermogenesis raises total body energy expenditure and reduces fat mass (Okamatsu-Ogura et al., 2020; Saito et al., 2020). In BAT, UCP1 is highly expressed and oversees adaptive thermogenesis. When UCP1 is stimulated, the respiratory chain is activated. The combustion of readily available substrates produces heat, which the circulatory system then transfers to the other parts of the body (Ricquier, 2011; Jorge et al., 2017). The UCP1 protein helps the body burn energy and lose weight through a process called thermogenesis. It is also involved in maintaining healthy levels of glucose and lipids in the body (Kozak and Anunciado-Koza, 2008; Stanford et al., 2013).

### 2.2 Dysregulated adipokine secretion

Adipokines are substances produced by fat cells that play a crucial role in the relationship between metabolism and immune system function. However, when they are not regulated properly, as is often the case in obesity, they can cause persistent, low-level inflammation that can lead to various health problems (Taylor, 2021). Adipose tissue has functions beyond just storing energy. It also produces and releases various substances called adipokines, which can act on the fat tissue itself as well as other organs like the heart and lungs to regulate their function. Adipokines act in both local and systemic ways (McGillis, 2005). In addition, adipokines can influence the production of cytokines, which are signaling molecules that can have either pro-inflammatory or anti-inflammatory effects, depending on the specific circumstances (Fu et al., 2017; Salvator et al., 2021; Palma et al., 2022). In general, the production of most adipokines increases in obesity, and the pro-inflammatory ones tend to contribute to the development of health problems associated with obesity as well as in lean subjects, such as asthma where adipokines play an important role in the pathogenesis (Sood and Shore, 2013).

#### 2.2.1 Proinflammatory adipokines

##### 2.2.1.1 Leptin

Leptin is the most well-known proinflammatory adipokine, and it was originally described as a satiation hormone (Friedman and Halaas, 1998). Leptin stimulates the production of chemokine (C-C motif) ligand 2 (CCL2) and vascular endothelial growth factor in human hepatic stellate cells and activates monocytes and macrophages to produce pro-inflammatory interleukin-6 (IL-6), tumor necrosis factor-alpha (TNF-α), and interleukin-12 (IL-12) (Gainsford et al., 1996; Aleffi et al., 2005). Other inflammation signals, such as TNF-α and lipopolysaccharides (LPS), increase leptin and leptin receptor expression (Grunfeld et al., 1996; Gan et al., 2012). In CD4^+^ T cells, leptin also increases the production of pro-inflammatory Th1 cytokines while suppressing the release of anti-inflammatory Th2 cytokines such as interleukin-4 (IL-4) (Lord et al., 1998). Increased expression of leptin is reported to be associated with obesity-asthma phenotype through the activation of the STAT3 signaling pathway (Chong et al., 2019). Several clinical studies reported an increased level of leptin in obese asthmatics as compared to the non-obese population (Sánchez-Ortega et al., 2022). As reported in human normal BEAS-2 bronchial epithelial cells, leptin promotes exacerbation in obese asthmatic patients by upregulating the mitochondrial reactive oxygen species/NOD-, LRR-, and pyrin domain-containing protein 3 (mostly known as mtROS/NLRP3) inflammasome signaling pathways (Chong et al., 2021). A recent systematic review and meta-analysis also confirmed the pathological role of leptin in obesity-associated asthma where leptin was reported to hold responsible for activating the signaling pathways of inflammation and parasympathetic system that lead to the disease severity (Sánchez-Ortega et al., 2022).

##### 2.2.1.2 Interleukin-6

In adult adipose tissue, IL-6 is highly expressed and favorably linked with obesity (Kwon and Pessin, 2013). One of the most researched factors linked to poor adipogenesis and insulin resistance is IL-6, a pro-inflammatory and immunomodulatory cytokine. IL-6 suppresses the synthesis of adiponectin and impairs insulin signaling, which together contribute to inflammation in obesity (Tzanavari et al., 2010). C-reactive protein (CRP) is also produced as a result, which intensifies the inflammatory response (Bahceci et al., 2007). Additionally, IL-6 participates in the early and late phases of the asthma response and is correlated with the severity of the condition (Sideleva et al., 2012). Plasma IL-6 exerts its pathological aspects in obese-asthmatics by targeting several lung cells, i.e., endothelial cells, epithelial cells, and other airway cells as well as immune cells like regulatory T cells and Th17 cells. The same study reveals that IL-6 high subset of obese asthmatics suffering from other metabolic dysfunctions like hypertension has the highest incidence of asthma as compared with obese asthmatics without any metabolic dysfunctions. This finding reveals a contentious relationship between high plasma IL-6 and obese asthmatics with other metabolic dysfunctions that warrant further studies (Peters et al., 2016).

##### 2.2.1.3 Tumor necrosis factor

Tumour necrosis factor-alpha (TNF-α) is a multi-functional cytokine that can regulate many cellular and biological processes such as immune function, cell differentiation, proliferation, apoptosis, and energy metabolism. It is synthesized as a 26-kDa transmembrane monomer (mTNF-α). In the pulmonary environment, alveolar macrophages can create significant levels of TNF-α, which plays an important role in the pathogenesis of asthma (Kim et al., 2012). The TNF-α receptor 1 (TNFR1) mediates the anti-adipogenic effects of TNF-α (Cawthorn and Sethi, 2008). The number of preadipocytes undergoing differentiation in the abdomen subcutaneous tissue is decreased due to elevated levels of mitogen-activated protein kinase kinase 4 (MAP4K4), which is engaged in the TNF-signaling pathway, leading to hypertrophic fat cells in conjunction with obesity. This implies that fat accumulation and proinflammatory capability are inversely correlated (Cawthorn et al., 2007; Isakson et al., 2009; Al-Mansoori et al., 2022). A study conducted on diet-induced obese (DIO)-mice revealed that controlling obesity through exercise and diet control was found to be an effective means decrease the pulmonary TNF-α levels which result in decreased asthma severity in obesity-associated asthma (Kim et al., 2015). Extracellular matrix (ECM) protects the airways by preventing them from collapsing during expiration, and metalloproteinase (MMPs) enzymes are responsible for ECM renewal, making them important in pulmonary biology. In obese mice, intraperitoneal administration of anti-TNF- monoclonal antibody significantly decreased lung matrix metallopeptidase 9 (MMP-9) activity, demonstrating a link between TNF- and increased asthma severity in obese subjects (Vieira et al., 2020).

##### 2.2.1.4 Resistin

Resistin, a 10 kDa polypeptide of 114 amino acids in rodents, is known to produce pulmonary inflammation and insulin resistance (Holcomb et al., 2000; Steppan et al., 2001). Peripheral blood inflammatory cells, monocytes, and macrophages are the principal producers of resistin. Resistin has also been found in the cells of the bone marrow, lungs, placenta, pancreatic islet tissue, and adipose tissue (Le and Pang, 2006). Resistin expression in human macrophages is induced by inflammatory cytokines such IL-1, IL-6, TNF-α, and LPS. Through the NF-κB signalling pathway, resistin induces human peripheral mononuclear cells to release IL-6 and TNF-α, whereas rosiglitazone, a peroxisome proliferator-activated receptor (PPAR) agonist, inhibits resistin expression in adipose tissues, reducing inflammatory responses (Bokarewa et al., 2005). As per a recent study, serum resistin levels were found to be elevated in obese asthmatics especially in females as compared to males which further confirms the role of resistin in the exaggeration of asthma in obese (Hosny et al., 2021). The hResistin:adiponectin ratio is found to be much higher in obese asthmatics, supporting the idea that hResistin may make a significant contribution to increased severity in the obese population which encourages the idea that this ratio could be a possible therapeutic avenue for managing the obese asthma phenotype (Lin and Johns, 2020).

There are several other proinflammatory adipokines that have been suggested to contribute to the inflammation associated with obesity, although they have not been studied as much as others. These include chemerin, retinol binding protein 4 (RBP4), CC-Chemokine Ligand 2 and CC-Chemokine Receptor Type 5, Angiopoietin-Like Protein 2, and lipocalin 2 (LCN2).

#### 2.2.2 Anti-inflammatory adipokines

##### 2.2.2.1 Adiponectin

Adipocytes strongly express the potent anti-inflammatory molecule adiponectin. A complex molecule, adiponectin can aggregate into complexes with low, intermediate, and high molecular weights in the blood. The AdipoR1 and AdipoR2 receptors, which are involved in immune cells and tissues, activate AMP-activated protein kinase (AMPK) to produce its effects (Yamauchi et al., 2003). The high-molecular-weight complex possesses anti-inflammatory effects that are known to suppress inflammation by preventing NF-κB activation and decreasing cytokines including TNFα, IL-6, and IL-18 (Yokota et al., 2000; Ouchi et al., 2011). Increased inflammatory responses are seen in adiponectin-knockout mice, pointing to the crucial role of adiponectin in reducing tissue and systemic inflammation (Summer et al., 2008; Yoshida et al., 2014). Many clinical investigations have discovered lower serum adiponectin levels in adult obese-asthmatic patients when compared to non-obese asthmatics or controls, with a statistically significant connection between BMI and adiponectin (Bianco et al., 2017). Low adiponectin level is especially found in female obese asthmatics as compared to men which imply that adiponectin responds differently in both sexes having a common type of asthma in obese phenotype (Sood et al., 2008). It has recently been revealed that a high BMI and a low level of adiponectin in children may suggest severe asthma (Machado et al., 2022). Although there have been reports of the protective effect of adiponectin on obese asthmatics, however, there are also reports of the negative impacts of adiponectin in the same condition. As per reports, an elevated level of serum adiponectin is linked to adverse clinical outcomes of asthma in men but not in women, however, the study ruled out the association of BMI with the change in absolute forced expiratory volume in 1 s (FEV1) (Sood et al., 2011). The controversies surrounding adiponectin may be due to polymorphisms in the ADIPOQ gene, where rs822396 and the T allele of rs1063537 have been linked to an increased risk of asthma, whereas variants of rs11760956, rs11763517, and rs2167270 have been linked to a protective nature in obese-asthmatics. There is an urgent need to clarify the diverse role played by adiponectin in different asthma and obese phenotypes before targeting adiponectin for therapeutic implications.

##### 2.2.2.2 C1q/TNF-related protein (CTRP) family

A highly conserved family of adiponectin paralogs known as the C1q/TNF-related proteins (CTRP3) is reported to play an important role in obesity-associated comorbidities (Wong et al., 2004). Similarly, to adiponectin, some CTRPs such as CTRP6, CTRP9, and CTRP12 are mainly expressed in adipose tissue (Wong et al., 2008). CTRP3 is reported to inhibit PS-induced expression of macrophage migration inhibitory factor (MIF), MCP-1, and C-C motif chemokine ligand4 (CCL4) in human monocyte-derived macrophages (Weigert et al., 2005). CTRP6 is reported to increase the expression of anti-inflammatory cytokine IL-10 in human monocyte-derived macrophages through the p42/44 mitogen-activated protein kinase (MAPK)-dependent pathway (Kim et al., 2010). Plasma CTRP9 levels are reported to decrease in mouse models of obesity. Additionally, CTRP9 forms heterotrimers with adiponectin and shares the receptor Adipo R1 with adiponectin in vascular endothelial cells and cardiac myocytes (Kim et al., 2010; Kambara et al., 2012; Ohashi et al., 2014). CTRP12 or adipolin is an insulin-sensitizing adipokine that is abundantly produced by AT and whose expression levels decrease in rodent models of obesity (Enomoto et al., 2011). Additionally, adipolin treatment reduced macrophage infiltration and the expression of pro-inflammatory genes in the adipose tissue of obese mice (Alzaim et al., 2020). In a clinical study involving chronic obstructive pulmonary disease (COPD) patients with a BMI of 22.88 ± 2.78 kg/m^2^, CTRP-3 and CTRP-5 were found to be elevated in COPD patients however only CTRP-5 was inversely related to FEV1/FVC ratio. The study discovered that CTRPs were statistically unrelated to adiponectin levels, however, like CTRPS, adiponectin was found to be elevated in COPD patients (Li et al., 2015). The elevated level of adiponectin might be due to COPD-induced hyperinflation, necessitating chronic respiratory muscle exercise to restore normal lung function (Pathak et al., 2020) but the reason behind the elevated expressions of the CTRPs is yet to divulge. Although, there are reports of the effect of CTRPs in obese conditions and in pulmonary conditions like COPD, however, there has been a minimal report on the role of CTRPs in modulating obesity-associated asthma.

##### 2.2.2.3 Omentin

Omentin is a type of adipokine whose levels tend to be lower in obese individuals and are negatively correlated with a measure of arterial stiffness (Shibata et al., 2011; Yoo et al., 2011). Additionally, coronary artery disease prevalence and angiographic severity are adversely correlated with omentin expression (Askin et al., 2020). Omentin increases eNOS activity and reduces TNFα-induced endothelial COX2 expression (Zhong et al., 2012). Omentin-1 has been demonstrated to reduce inflammatory responses in endothelial cells in culture by inhibiting JNK activation via the AMPK/eNOS signalling pathway (Yamawaki et al., 2011). Reports on the role played by omentin in respiratory diseases are inconsistent. Omentin was found to be elevated in the airway epithelial sample of asthma as well as in the circulatory sample of obstructive sleep apnea syndrome and decreased in the airway epithelium sample of COPD (Zhou et al., 2017). A significantly reduced level of circulating omentin was found in a group of none-obese (BMI 23.81 ± 3.57 kg/m^2^) patients with severe persistent asthma as compared to the control group which revealed the inverse relationship of omentin to asthma (Zhou et al., 2018). There has been reporting on the decreased level of omentin in obese subjects as well as in non-obese asthmatics, however, a study on the level of omentin in obese asthmatics is not missing in the scientific literature which is the need of the hour to ascertain the role of omentin the later condition.

### 2.3 Activation of NLRP3 inflammasomes and macrophage polarization

Innate immunity depends on pathogen-associated molecular patterns (PAMPs) and danger-associated molecular patterns (DAMPs), which activate a variety of immune cells including dendritic cells and the inflammasome (Lee et al., 2014). Inflammasomes are multi-protein complexes that are essential for innate immunity and play a role in controlling inflammatory responses that are triggered by infection or cellular injury. There are numerous different types of inflammasomes, including NLRC4, NLRP1, NLRP6, AIM2, and IFI16 (Rathinam et al., 2012), NLRP3, however, is the inflammasome that has received the most attention. The NLRP3 inflammasome is a group of proteins found in immune cells such as macrophages and dendritic cells, as well as other types of cells. The activation of NLRP3 has also been linked to various metabolic and inflammatory conditions such as gout, diabetes, insulin resistance, and obesity. The activation of NLRP3 plays a crucial role in various metabolic conditions however, too much activation of the inflammasome can cause excessive inflammation and damage to tissues, contributing to the development of chronic inflammatory diseases such as asthma (Williams et al., 2021). The activation of inflammasome-mediated reactions is necessary for an appropriate immunological reaction; however, excessive activation can lead to elevated inflammation and tissue damage, affecting the pathogenic mechanisms of chronic inflammatory diseases such as asthma. In a recent study, activation of NLRP3 inflammasome gene expression along with other inflammatory markers like TLR4, and IL-1β were found to exaggerate the asthmatic episode in obese asthmatic patients as compared to non-obese asthmatics which confirms the negative role of NLRP3 in obesity-associated asthma (Wood et al., 2019). The relationship between NLRP3 and asthma was further cemented when the activation of NLRP3 was inhibited by some novel NLRP3 inhibitors or by silencing of NLRP3. There have been reports of reduction of airway hyperresponsiveness (AHR) and airway inflammation in murine models of ovalbumin (OVA)-induced respiratory infections model by NLRP3 inhibitors like MCC950 and AC-YVAD-cho (Kim et al., 2017). Similarly, silencing the NLRP3 gene through short hairpin RNA is reported to reduce NLRP3 mediated IL-1β secretions in human bronchial epithelial cells (Theofani et al., 2019). Macrophages are immune cells that play a role in inflammation and the immune response. The activation state of macrophages, or their “polarization,” has been found to significantly influence the development of asthma. When macrophages are recruited to a specific area of the body, they can be activated in one of two ways: a “classically activated” or M1 state, or an “alternatively activated” or M2 state, depending on the local environment (Saradna et al., 2018). Weisberg et al. (2003) and Xu et al. (2003) first described the infiltration of a significant number of macrophages into the adipose tissues of obese persons. M1 macrophages typically produce inflammation through the activation of receptors for TNF-α, IL-1β, and CD36, which leads to the activation of NF-κB and the production of NLRP3 and other pro-inflammatory cytokines such as IL-1β and IL-18. This process, which is known as “priming,” sets the stage for further inflammation (Russo and Lumeng, 2018). However, the NLRP3 inflammasome requires a second “hit” or stimulus to assemble and become active. This second stimulus is usually provided by substances such as ceramides, fatty acids, oxidized low-density lipoproteins, and cholesterol crystals, which bind to TLR 2/4 receptors (Rajamaki et al., 2010; Camell et al., 2015; Karasawa et al., 2018; Okla et al., 2018). The claim of the second “hit” by substances for NLRP3 activation was further cemented when microarray data demonstrated a significant increase of ceramide accumulation during M1 macrophage polarization in obese asthmatics. In the same study, M1 macrophage polarization as well as an elevated level of airway hyperresponsiveness (AHR) and C18:0 ceramide levels were observed in obese mice which confirms the role of ceramide or similar substances in the deterioration of obesity-associated asthma (Choi et al., 2020). Finally, sustained activation of NLRP3 leads to the formation of GSDMD pores in the membrane of macrophages (Liu et al., 2016). This process, called “pyroptosis,” causes the cell to die and release pro-inflammatory molecules into the surrounding area. It does this by disrupting the osmotic potential inside the cell (Evavold et al., 2018; López-Reyes et al., 2020). M1 macrophages are believed to be beneficial in asthma and make a significant contribution to the pathogenesis of obesity, whereas M2 macrophages are responsible for asthma pathology but restrict the obesity comorbidities (Sharma et al., 2018). T2 cytokines and the NLRP3 inflammasome, which is responsible for obesity-related asthma, were recently discovered to have a link, making them targets for therapeutic applications (Pinkerton et al., 2022).

### 2.4 Dysregulated melanocortin pathway

Eosinophil infiltration into the airways, a characteristic of bronchial asthma, is crucial to the pathophysiology of the disease (De Monchy et al., 1985; Bousquet et al., 1990). Although there have been significant advancements in our understanding of the aetiology of this disease over the past 20 years, the therapy of choice is still the same as it was 30 years ago: inhaled 2-adrenoceptor agonists and glucocorticoids. As a result, there is still a very real medical need for treating the disease’s symptoms and underlying pathology. Melanocortins may be used to suppress airway inflammation, according to one study. In an OVA-challenged model of airway inflammation, α-Melanocyte-stimulating hormone (α-MSH) reduced eosinophil migration and subsequently IgE, IL-4, and IL-13 levels, but had no effect on IL-5. Although it may appear odd that eosinophils are inhibited but not IL-5, α-MSH inhibits VCAM-1, which is regulated by IL-4 and IL-13, which may account for the decrease in migratory cells. α-MSH can also promote the anti-inflammatory cytokine IL-10, as was previously mentioned with the production of the anti-inflammatory protein heme oxygenase-1 (HO-1) (Lam et al., 2005). Given that α-MSH is inactive in IL-10 null animals, this would seem to be a crucial mechanism for the anti-inflammatory effects of the melanocortin. Very few substances can treat the underlying pathophysiology of bronchial hyperresponsiveness, even though many have been demonstrated to block certain components of the inflammatory response associated with asthma. Since α-MSH suppressed the inflammation as well as cellular migration and cytokine production, it had steroid-like effects (Montague and O’Rahilly, 2000). The central melanocortin system is reported to play important role in obesity and diabetes. Aside from its effects on food intake and body weight, α-MSH has anti-inflammatory activities in both immune cells and non-immune cells (e.g., adipocytes) that express melanocortin receptors which may be a potential target for obesity-associated asthma (Goit et al., 2022).

### 2.5 Depletion of ROS regulator

The rise in free fatty acid levels boosts adipocyte ROS production by activating NADPH oxidase and decreasing the expression of enzymatic antioxidants, which is correlated with fat adipocyte fat storage (Hattori et al., 2005; Johnson et al., 2007; Devaraj et al., 2008). Anti-inflammatory adiponectin levels decrease and the concentration of pro-inflammatory adipocytokines increases in adipocytes with OS (Soares et al., 2005; Chen et al., 2009). Because there are no accurate ways to measure oxidative stress *in vivo*, most of the evidence indicating the action of ROS in asthma is indirect or circumstantial. The analysis of lipid peroxidation based on diene conjugate and thiobarbituric acid (TBA), measurement of increases in nitric oxide (NO), H_2_O_2_, and pentane in exhaled gas or breath condensates, and evaluation of substrate oxidisability (or spin trapping) of free radical adducts *ex vivo* all have low sensitivity and specificity. However, utilizing these techniques, it was discovered that adults and children with different asthma severity and acute asthma exacerbations had an increased generation of ROS (Jarjour et al., 1992; Dohlman et al., 1993; Antczak et al., 1997; Olopade et al., 1997; Saleh et al., 1998). Nrf2 is a key regulator in the transcriptional activation of antioxidation and biosynthesis of endogenous antioxidants (Kasai et al., 2020). Nrf2 signals the nucleus during oxidative stress to promote the expression of HO-1, which in turn, protects the lung from oxidative stress (Pathak et al., 2021). Both Nrf2 and HO-1 are reported to be downregulated in obese conditions.

### 2.6 Notch signaling pathway

Currently, Notch signaling pathway has gained considerable attention as a therapeutic target for obesity-associated asthma. Notch signaling activation is reported to inhibit adipocyte differentiation by suppressing the expression of CCAAT/enhancer binding protein alpha (C/EBPα) and peroxisome proliferator-activated receptor gamma (PPARγ) in the 3T3-L1 preadipocytes (Ross et al., 2004). In obese mice, inactivating Notch1 causes the browning of white adipose tissue and increased expression of UCP1, a key regulator of thermogenesis (Bi et al., 2014). Notch signaling pathway having CD4^+^ T cell (Th17 cell) is reported to express increased interleukin (IL)-17 in obese asthmatic individuals. In a preclinical study, *γ*-Secretase inhibitor (GSI) is reported to reduce the Notch1, Th17 cell proportion and serum IL-17 in a diet-induced-obesity-ovalbumin (DIO-OVA) C57BL/6 mice model of asthma (Zeng et al., 2019). Pre-clinical and clinical studies involving Notch signaling pathway reveals that targeting Notch signaling pathway therapeutically may open new avenues in the treatment of obesity-associated asthma in recent times.

### 2.7 Gut microbiota dysbiosis

Investigating how gut microbiota affects asthma has gained popularity in recent years. Children at risk for developing asthma exhibited considerably reduced relative abundances of the taxa *Faecalibacterium*, *Lachnospira*, *Rothia*, and *Veillonella* (FLVR) in their gut microbiomes at 3 months of age. Increased risk of asthma was linked to higher *Veillonella* abundance and lower *Roseburia*, *Alistipes*, and *Flavonifractor* abundances at 1-year-old age for children with asthma at age 5 (*n* = 60), albeit the association was only significant for kids whose mothers also had asthma (Stokholm et al., 2018). According to various research, people with asthma had higher concentrations of *Streptococcus pneumoniae*, *Haemophilus influenzae*, *Moraxella catarrhalis*, and *Haemophilus* species in their airway microbiomes (Bisgaard et al., 2007; Hilty et al., 2010; Green et al., 2014). Diet can cause fast microbial alterations in the gut microbiome. According to one study, lifetime vitamin D administration increased the number of *Acinetobacter* operational taxonomic units (OTUs) in the lungs of female mice. The lung microbiota was significantly more altered by allergic airway illness that was brought on by ovalbumin, suggesting that vitamin D’s ability to modulate the microbiota may be constrained (Roggenbuck et al., 2016). According to recent studies, food, gut microbiota, and airway inflammation interact via short chain fatty acids (SCFAs) and pro- or anti-inflammatory cytokines. Additionally, because observational human studies are associative, creating possible prebiotic or probiotic treatments might benefit from employing mouse models that focus on the causative relationships between microbiome dysbiosis and airway pathobiology. Obesity is linked to changes in the gut microbiota (Yuan et al., 2018). According to several studies, obesity in humans is linked to a higher *Firmicutes*/*Bacteroidetes* (F/B) ratio at the phylum level (Rahat-Rozenbloom et al., 2014; Serena et al., 2018). Which bacteria are most impacted by obesity has been the subject of widely varying studies. Lachnospiraceae, *Prevotellaceae*, *Megamonas* spp., *Roseburia* sp., *Veillonellaceae*, *Lactobacillus* spp., *Bacteroides fragilis*, *Faecalibacterium* sp., and a *Prevotella* spp. -dominated enterotype are more prevalent in people with obesity who have microbiota dysbiosis. Additionally, obesity was linked to decreased gene richness, decreased microbiota diversity, and decreased levels of Oscillospiraceae, *Succinivibrio* sp., Odoribacteraceae, Clostridaceae, and *Oscillospira* sp. (Murugesan et al., 2015; Liu et al., 2017). Significant correlations between changes in metabolic syndrome markers and gut microbial dysbiosis have been found in several human cohort studies. After adjusting for age and sex, Fu et al. (2015) found a negative correlation between gut bacterial richness and both BMI and serum triglyceride levels and a positive correlation between gut bacterial richness and serum HDL levels. According to this study’s estimates, the makeup of the gut microbiota may account for up to 6% of the variation in independent of age, genetic variables, blood lipid levels, and sex. The gut microbiome affects not only obesity and metabolic syndrome, but also airway inflammation mostly through the production of short-chain fatty acid and cytokine levels and triggers asthma (Kim, 2021). The administration of prebiotics and probiotics are reported to play an important role in the amelioration of obesity-associated asthma through modulation of the gut microbiota. In a recent study, dietary fiber pectin, a prebiotic, demonstrated a promising effect on the O_3_-exposed db/db mice model of AHR indicating the importance of targeting gut microbiota in the therapeutic strategy of obesity-associated asthma (Tashiro et al., 2019).

## 3 Bioactive phytoconstituents in obesity-associated asthma

Obese patients with asthma experience poor asthma control for many reasons, but a crucial one appears to be that their reaction to controller medication is impaired. Many studies have indicated that obese individuals do not respond as effectively to conventional control regimens. It appears that inhaled corticosteroids and combination inhalation corticosteroid-long-acting agonists are superior to montelukast for treating asthma in obese persons (Park et al., 2009). All existing approved asthma drugs were developed using lean animal models of asthma and tested on patient populations that were far slimmer than the typical patient population seen today (Halliwell et al., 1992). Herbs have always been a crucial and valuable treatment option for many persistent health problems, including obesity. Herbs generally have fewer side effects than single-compound drugs, except for allergic reactions in those particularly sensitive to them (Park et al., 2009). There has been a rise in scientific studies assessing the value of substances obtained from herbal medicine, which have traditionally been employed in folk medicine. Plant-based compounds, such as flavonoids, alkaloids, and terpenoids, have been shown to have biological effects *in vitro* and *in vivo*. *Tripterygium wilfordii*, *Hibiscus sabdariffa, Ilex paraguariensis, Coffea arabica, Caralluma fimbriata, Panax ginseng,* plants from solanaceae family, and many others have all been shown to have positive effects on obesity through various mechanisms, such as appetite suppression, triglyceride reduction, metabolic rate increase, pancreatic lipase inhibition, and so on. Therefore, they aid in the process of losing weight (MacNee, 2001; Kannaiyan et al., 2011).

**FIGURE 2:**
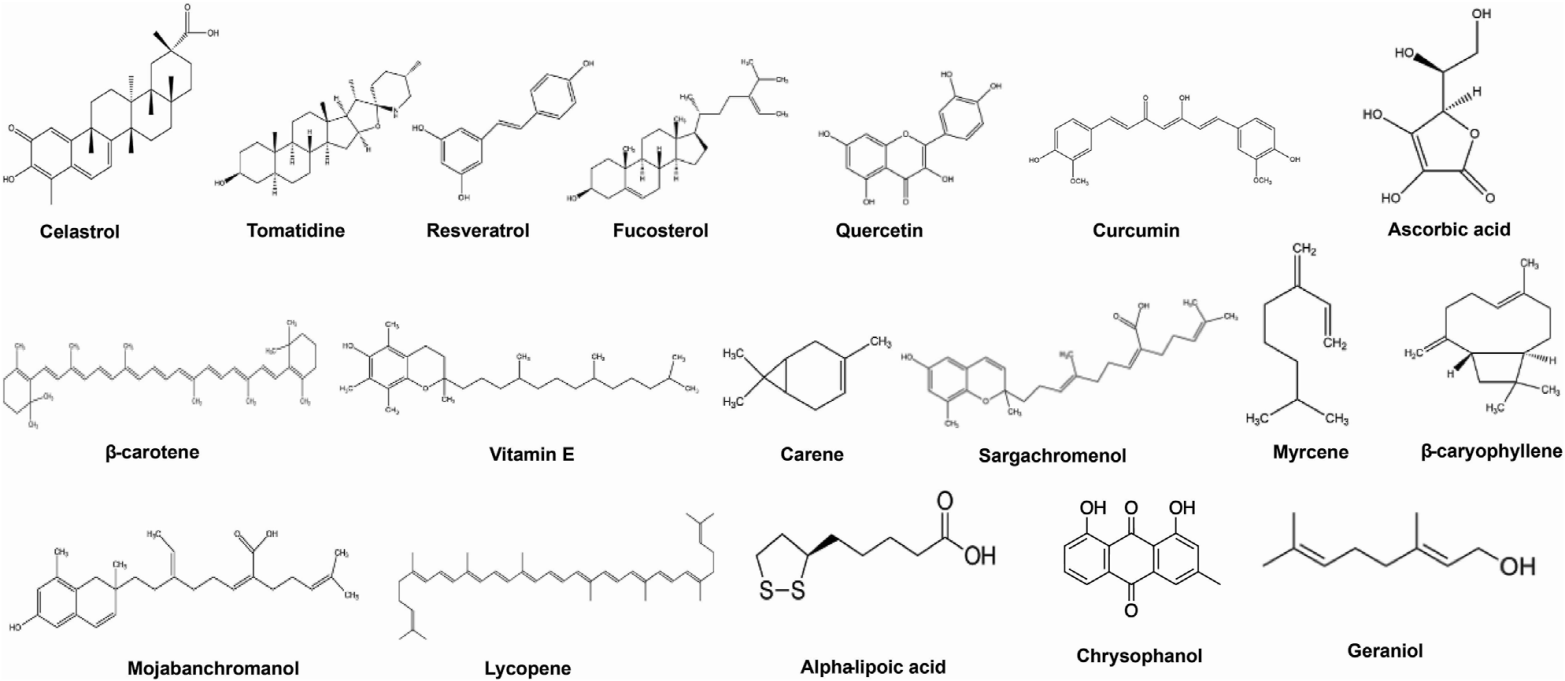
Bioactive phytoconstituents targeting obesity-associated asthma.

Here we have emphasized the effects of some important bioactive compounds like celastrol, tomatidine, resveratrol, quercetin, ascorbic acid, *β*-Carotene, chrysophanol, etc. (Figure 2), for treatment of obesity-associated asthma in experimental models and in humans and pointing out some possible mechanisms of action are emphasized.

### 3.1 Celastrol

Celastrol is an effective natural bioactive component isolated from *T. wilfordii*’s roots. It has proven highly beneficial in anti-inflammatory, anti-cancer, anti-rheumatic, and autoimmune illness (Kannaiyan et al., 2011). In addition, it has been shown to have a remarkable effect on body mass reduction in mice (Lee et al., 2006). Celastrol was reported to cause significant weight loss in a hyperleptinemic DIO mice model through the reduction of food intake, increasing energy expenditure, and leptin sensitivity (Liu et al., 2015). Celastrol has also been shown to lower airway inflammation and AHR in allergic asthma, which is interesting. On a model of obesity and asthma, celastrol therapy reduced the frequency of Th17 cell growth and IL-17A production in the lung and serum. The research study’s findings suggested that Th17 and its cytokine, as assessed in the spleen and lung, were strongly linked to AHR. In addition, celastrol inhibits Th17 in obese asthmatic mice, which has been demonstrated to decrease AHR. Celastrol reduces AHR and inhibits Th17 responses in obese asthmatic mice (Zhang et al., 1990).

### 3.2 Tomatidine

Tomatidine is a steroidal alkaloid under the *Solanaceae* family is obtained from leaves of eggplant, potato, and tomato. *Solanaceae* plants release tomatidine and *α*-Tomatine to combat fungal infections (Norton, 1998). Tomatidine has demonstrated promising anti-obesity in animal models. In male C57BL/6 mice, tomatidine significantly reduces body weight, fat weight, and regulates other metabolic parameters such as total cholesterol, fasting blood glucose, low-density lipoprotein, and triglyceride levels in serum (Wu et al., 2021). Tomatidine suppresses the reproduction of *Staphylococcus aureus*, according to a previous study. Additionally, tomatidine reduces the invasion activity of A549 human lung adenocarcinoma cells and promotes death in HL60 human myeloid leukemia cells. Tomatidine inhibits iNOS and COX-2 production by blocking the NF-κB pathway in LPS-stimulated RAW 264.7 cells, according to a study conducted (Steel and Drysdale, 1988). The research found that tomatidine ameliorates the allergic inflammatory response in the airways in asthma by reducing the levels of Th2-associated cytokines, goblet cell hyperplasia, eosinophil infiltration, and AHR. Tomatidine inhibited the generation of Th2 cytokines in bronchoalveolar lavage fluid. In lung tissue, tomatidine inhibited the expression of inflammatory and Th2 cytokine genes. Tomatidine decreased the generation of proinflammatory cytokines and CCL11 in inflammatory BEAS-2B bronchial epithelial cells *in vitro*. These findings suggest that tomatidine ameliorates AHR and eosinophil infiltration in asthmatic mice by inhibiting the inflammatory response and Th2 cell activity (Kuo et al., 2017). The above reports showcase the ameliorative action of tomatidine against both obesity and respiratory diseases like asthma and AHR by targeting various pathways which support the possible therapeutic efficacy of tomatidine against obesity-associated asthma which is yet to explore.

### 3.3 Curcumin (diferuloylmethane)

Curcumin, the active phytoconstituent in turmeric, is a polyphenol having anti-inflammatory, anti-amyloid, antibacterial, anti-tumor, anti-allergic, and anti-oxidative activities (Kurup and Barrios, 2008). Curcumin has been shown to have potent anti-obesity properties. Curcumin reportedly interacts with white adipose tissue to reduce chronic inflammation, macrophage infiltration, and nuclear factor B (NF-κB) activation. Curcumin also reduces the proinflammatory adipokines tumour necrosis factor (TNF), monocyte chemoattractant protein-1 (MCP-1), and plasminogen activator inhibitor type-1 (PAI-1) and increases adiponectin expression (Bradford, 2013). Curcumin acts as an HDAC activator or inhibits histamine release from mast cells, which reduces the production and level of cytokines like IL-2 and IL-5 in lung tissue (Biswas and Rahman, 2008). Curcumin’s ability to regulate asthma phenotypes indicates that it can alleviate asthma symptoms, decrease eosinophil recruitment to the airway, and improve airway hyper-responsiveness, all of which contribute to weight loss. These results suggest curcumin could be an effective adjunct treatment for obesity-related asthma (Brouet and Ohshima, 1995; Mortellini et al., 2000).

### 3.4 Resveratrol

Resveratrol (3,4,5-trihydroxystilbene) is a phytoalexin present in plant sources, such as grapes, berries, and peanuts as well as known as a component in red wine. Resveratrol is one of the polyphenolic molecules comprised of two phenol rings joined by a 2-carbon methylene bridge with multiple effects on cardiovascular protection, anticancer impact, and as a positive regulator of several areas of metabolism. Resveratrol can eliminate intracellular reactive oxygen species by activating and stabilizing antioxidant enzymes such as catalase, SOD, and glutathione peroxidase hemoxygenase (Das and Das, 2007; Shankar et al., 2007). In addition to its reducing properties, resveratrol has been shown to reduce inflammation by inhibiting prostaglandin production and decreasing the phosphorylation of ERK1/2, cyclooxygenase-2 activity, and the activity of various transcription factors, including NF-B, STAT3, HIF-1α, and *β*-Catenin (Shankar et al., 2007; Pirola and Fröjdö, 2008). Resveratrol is reported to exert its anti-obesity effect by the browning of WAT as well as by targeting several intracellular components such as sirtuin-1 (SIRT-1), adenosine monophosphate-activated protein kinase (AMPK), and the peroxisome proliferator-activated receptor coactivator-1 (PGC-1) (Price et al., 2012). Resveratrol can also modulate the innate immune response by inhibiting the expression of costimulatory molecules (CD80 and CD86) and major histocompatibility complex classes I and II in bone marrow-derived dendritic cells and by inhibiting angiogenesis pathway mediated by the expression of MMPs, VEGF, cathepsin D, ICAM-1, and E-selectin. Resveratrol has demonstrated potent ameliorative in obesity-associated asthma effect in experimental animal model. Resveratrol was effective in combating ovalalbumin and obesity-induced oxidative stress in obese asthmatic rat models by inhibiting Keap1 and activating the Nrf2 antioxidant defense system (Li et al., 2018).

### 3.5 Carotenoids

Carotenoids are a group of pigmented compounds produced by fruits, vegetables, and microorganisms. Carotenoids, which are found as micro-components in fruits and vegetables, are responsible for their yellow, orange, and red colours (Rao and Rao, 2007). *β*-Carotene, *α*-Carotene, lycopene, *β*-Cryptoxanthin and lutein are some of the major dietary carotenoids. In several observational studies, low level of *α*-Carotene, *β*-Carotene, and *β*-Cryptoxanthin has been linked to both asthma as well as obesity (Tobias et al., 2019). Total vitamin A is composed of preformed vitamin A (retinol) and provitamin A molecules, known as carotenoids, with beta-carotene being the most essential. Plants and microbes have carotenoids as pigments (Rao and Rao, 2007). Various studies have revealed that carotenoids may protect or inhibit some types of cancer, atherosclerosis, immunological problems, asthma, and other diseases (Gallicchio et al., 2008; Young et al., 2008). Carotenoids may influence the activation of many transcription factors (Niles 2004). *β*-Carotene inhibits oxidative stress-induced activation of NF-κB and generation of IL-6, TNF-α, and inflammatory cytokines in cells treated with *β*-Carotene. Carotenoids may impact the apoptotic process in healthy cells. *β*-Carotene can boost the expression of the anti-apoptotic protein Bcl-2 in normal cells but the pro-apoptotic protein Bax is downregulated when external stimuli are induced. It has also been demonstrated that lycopene regulates transcription factors. Lycopene inhibits AP-1 binding and reduces the activation of insulin-like growth factor-I in mammary cancer cells (Sharoni et al., 2004). These combined functions of vitamin A and dietary carotenoids make carotenoids an excellent anti-inflammatory agent in the treatment of obesity-associated asthma.

### 3.6 Vitamin C

Vitamin C (ascorbic acid) is a vital and effective antioxidant in the body’s aqueous environment. It exists in two interconvertible biologically active forms, ascorbic acid and its oxidized derivative, dehydro-ascorbic acid (Romieu and Trenga, 2001). As a hydrogen donor, vitamin C reverses oxidation and shields membranes from oxidation (i.e., reducing agent). Vitamin C, also known as ascorbic acid, has been shown to inhibit adipocyte lipolysis, regulate glucocorticoid release from the adrenal glands, inhibit glucose metabolism, modulate glucose metabolism, decrease glycosylation in obese-diabetic models, and reduce the inflammatory response (Garcia-Diaz et al., 2014). A deficiency in vitamin C is related to an increased risk of asthma in obese individuals, who have significantly lower levels of ascorbic acid; conversely, treatment with vitamin C is advantageous for asthmatic adults who smoke, lowering cough and wheezing (Allen et al., 2009). Vitamin C can also regulate factors that affect gene expression, apoptosis, and other cellular processes. It protects against cell death induced by a variety of stressors, and much of the protection is due to its antioxidant capacity (Romieu and Trenga, 2001). Vitamin C controls the AP-1 complex, which consists of the Fos and Jun superfamilies. Vitamin C treatment of cells exposed to UV-B resulted in a 50% decrease in JNK phosphorylation, which activates AP-1 and inhibits the JNK/AP-1 signaling pathways (Catani et al., 2001). Currently, however, the evidence from randomized controlled trials is insufficient to suggest a specific role for vitamin C in the treatment of asthma due to the various study design and typically flawed reporting systems (Kaur et al., 2009).

### 3.7 Vitamin E

There are eight distinct forms of fat-soluble vitamin E. It consists of a group of chemicals belonging to two closely related families, the tocopherols and tocotrienols, each of which exists in multiple isomeric forms (Riccioni and D’Orazio, 2005). Alpha-tocopherol is the most active form of vitamin E in humans and its primary antioxidant activity is lipid peroxidation prevention (Moriguchi and Muraga, 2000; Devarah et al., 2002). Vitamin E is also reported to ameliorative role in the obese condition. Vitamin E administration reduced oxidative stress, deposition of collagen in the visceral adipose tissue of obese mice, which resulted in a reduction in circulating cytokines and hepatic steatosis, hypertriglyceridemia, and followed by improvement of insulin sensitivity (Alcalá et al., 2015). Vitamin E is well known for playing a potent role in curbing the generation of free radicals which is due to its high concentration in the body among all lipid-soluble vitamins. Vitamin E is associated with a low incidence of asthma in infants as well as in adults. Vitamin E administration during pregnancy is associated with a lower chance of exacerbation of asthma in children (Miyazawa et al., 2019). Vit-E alleviated AHR by lowering Th2 responses, namely, as IL-4, IL-5, IL-13, and OVA-specific IgE, eotaxin, transforming growth factor-1, airway inflammation, lipid peroxidation, and nitric oxide metabolites, and by restoring mitochondrial dysfunction in the lungs (Mabalirajan et al., 2009). Besides, several epidemiological studies have demonstrated the effectiveness of vitamin E supplementation in treating bronchial asthma in obese and non-obese patients (Picado et al., 2001; Troisi et al., 1995; Trenga et al., 2001; Pearson et al., 2004; Pletsityi et al., 1995). However, the exact mechanism underlying the ameliorative role of vitamin E in obese asthmatics is yet to be unearth.

### 3.8 Alpha-lipoic acid

*α*-Lipoic acid (ALA), an organosulfur chemical produced from octanoic acid, is also known as thiothic acid. ALA is quickly absorbed from the diet and swiftly transformed into dihydrolipoic acid (DHLA), its reduced dithiol form. LA and DHLA are both potent antioxidants (Prior and Cao, 2000). Spinach, broccoli, and tomatoes are high in lipoyllysine -containing plant sources. ALA has been demonstrated to drastically lower serum IgE concentration, attenuate Th2 cytokines, IL-4, IL-5, IL-13, and IL-18, and reduce NF-B activation by decreasing intracellular ROS levels in a mouse model of asthma with allergic airway inflammation (Bustamante et al., 1998). In addition, ALA activation connected to VEGF expression, a crucial asthma factor, suppresses the pathogenesis of HIF-1 (Bustamante et al., 1998). These studies demonstrate that ALA is a multifunctional molecule in airway inflammatory illnesses, acting as a potent antioxidant and a regulator of gene expression that induces inflammatory cascades and plasma exudation and promotes the management of obesity (Cao and Phillis, 1995). ALA has demonstrated a marked effect in targeting obesity clinically by reducing body weight and BMI significantly without any effect on waist circumference (Namazi et al., 2018; Vajdi and Abbasalizad Farhangi, 2020). As observed in the animal models, ALA exerted its anti-obesity effect by modulating oxidative stress, and inflammation and by increasing insulin sensitivity in the subcutaneous and visceral adipose tissue of high-fat diet rats (Dajnowicz-Brzezika et al., 2022; Sztolsztener et al., 2022). ALA is reported to play a positive role in the antioxidant level of pulmonary tissue of obese asthmatic mice. The study reported that the administration of ALA combined with L-ascorbic acid to obese C57BL6 mice resulted in the upregulation of the endogenous antioxidant enzymes superoxide dismutase and catalase which demonstrated the antioxidant property of ALA. However, the effect of a single administration of ALA in obese asthmatic animals is reported to date.

### 3.9 Chrysophanol

Chrysophanol is an anthraquinone reported to found in many plants, insects, and microbes. In plants, chrysophanol was first reported in *Rheum rhabarbarum*, in fungi it was first reported in *Penicillium islandicum* Sopp, however, in insects, chrysophanol was reported to use as a defensive chemical (Yusuf et al., 2019). In animal models, chysophanol has demonstrated potential anti-obesity efficacy. Chrysophenol has been shown to reduce lipid accumulation in HFD rats’ primary hepatocytes, reduce inflammation by significantly lowering IL-6 and IL-1 levels, and increase IL-10 levels. Furthermore, the same study found that chrysophanol decreased the expression of lipogenic genes by activating AMP-activated protein kinase (AMPK)/Sirtuin 1. (SIRT1). Chrysophanol has also demonstrated ameliorative effects in asthma by inhibiting the activation of nuclear factor-kappa B (NF-kB) signaling pathway in animal models (Song et al., 2019). In a recent study involving the systematic pharmacological and bioinformatics analysis, chrysophanol was found to a potential candidate for combating obesity asthma/childhood asthma comorbidities. The study found that chrysophanol It was discovered that chrysophanol regulates inflammation by being involved in the resistance of EGFR tyrosine kinase inhibitor, the HIF-1 signaling pathway, and the trapping of extracellular neutrophils. However, the study emphasized the importance of experimental verification of their claim using *in-vitro*, *in-vivo*, and OMICS technologies (Liu et al., 2023).

### 3.10 Flavonoids

With more than 8,000 molecules reported, flavonoids are the most important group of low molecular weight polyphenolic secondary plant metabolites. They are found in fruits, vegetables, nuts, seeds, stems, flowers, roots, tea, wine, and coffee, and are prevalent in our diet (Hollman and Katan, 1999). Flavonoids limit the release of histamine and other granule-associated mediators by reducing the activation of basophils and mast cells (Harborne and Williams, 2000). Furthermore, flavonoids suppress the synthesis of IL-4, IL-13, and CD40 ligand, whereas they stimulate the production of novel phospholipid-derived mediators. Quercetin, one of the well-studied flavonoids, inhibits concentration-dependently the eosinophilic secretion of Charcot-Leyden crystal protein and eosinophil cationic protein (Balsano and Alisi, 2009). In addition, quercetin’s inhibitory effect on other inflammatory cells appears to be superior to that of any other clinically accessible drug (Middleton et al., 2000). Li et al. (2010) have proven that apigenin displays anti-inflammatory effects in a mouse asthma model and can modify the immune response to allergens toward the T-helper type 1 cell (Th1) profile. These results imply that flavonoids are excellent anti-allergenic and anti-inflammatory medicines for the treatment and prevention of asthma. Vascular alterations are one of the primary factors in the pathophysiology of asthma (Li et al., 2010). The alterations include a rise in vascular permeability, vascular dilation/engorgement, and vasculogenesis/angiogenesis (Williams and Grayer, 2004). Flavonoids and their related chemicals influence the production of HIF-1, VEGF, matrix metalloproteinases (MMPs), and the epidermal growth factor receptor, but also inhibit the NF-B, PI3K/Akt, and ERK1/2 signaling pathways (Hollman and Katan, 1999). Observations indicate that flavonoids and their related substances block some phases of angiogenesis, including cell migration, microcapillary tube formation, and matrix metallopeptidase (MMP) production.

### 3.11 Bioactive compounds from marine sources

Fucosterol, a phytosterol derived from the marine brown algae *Padina boryana*, exhibited anti-inflammatory properties via dose-dependently down regulating pro-inflammatory cytokines (IL-1, IL-6, and TNF) and the Nrf2/HO-1 pathway and aid in the process of losing weight in obesity associated asthma (Sohn et al., 2021). Mojabanchromanol (MC), a chromanol isolated from the brown alga *Sargassum horneri*, exhibited anti-oxidant effects via the attenuation of particulate matter-induced oxidative stress, the reduction of the ROS-mediated phosphorylation of MAPK extracellular signal-regulated kinase 1/2 (Erk1/2) and of c-JNK, and the inhibition of the secretion of pro-inflammatory cytokines (The authors proposed that mojabanchromanol be developed as a treatment for airway inflammation caused by particulate matter (Herath et al., 2020). Sargachromenol, isolated from *S. horneri*, exhibited anti-inflammatory effects in LPS-stimulated RAW 264.7 macrophages, by reducing nitric oxide (NO); and in intracellular reactive oxygen species (ROS), by reducing the mRNA expression levels of inflammatory cytokines (IL-1, IL-6, and TNF-) and by inhibiting the activation of NFκB and MAPK signaling (Herath et al., 2020). Pyrenocine A, which is derived from the marine-derived fungus *Penicillium paxilli*, suppresses pro-inflammatory mediators (TNF- and PGE2) and NFB-related gene expression in LPS-stimulated macrophages (Toledo et al., 2014). Spirulina extract (Immulina^®^), a high-molecular-weight polysaccharide extract from the Cyanobacterium *Arthrospira platensis* (Spirulina), exhibited anti-inflammatory and inhibitory effects on histamine release from RBL-2H3 mast cells and an induced allergic inflammatory response. Moreover, it has the potential to block the IgE-antigen-complex-induced generation of TNF-, IL-4, leukotrienes, and histamine, and it exhibited promising results in relieving allergic rhinitis symptoms (Appel et al., 2018).

### 3.12 Bioactive compounds from essential oils

Essential oils from *Citrus paradisi*, *Cymbopogon nardus* L., *Pogostemon cablin* Benth, *Hamelia patens*, *Citrus sinensis* and many more are reported to have promising anti-obesity effects. The anti-obesity effects in those essential oils might be due to the presence of a myriad of bioactive phytoconstituents like *α*-Pinene, *β*-Myrcene, geraniol, *β*-Citronellol, citronellol, *α*-Patchoulene, *β*-Patchoulene, carvacrol, linalool, D-limonene (De Blasio et al., 2021; Miya et al., 2021). Along with their anti-obesity potential, these essential oil isolated bioactive phytoconstituents has demonstrated significant efficacy in respiratory diseases like asthma and AHR. Geraniol is reported to ameliorate allergic asthma by reducing eosinophils, Th2 cytokines like IL-4, 5 and 3 and increasing Th1 cytokine like interferon γ, Nrf2 protein expression, and reduced glutathione (Xue et al., 2016). *β*-Caryophyllene (BCP), a dietary bicyclic sesquiterpene cannabinoid-2 (CB2) receptor-ligand is repoeted to found mainly in the essential oils of *Syzygium aromaticum* (Prashar et al., 2006). BCP suppresses obesity-related AHR by its effects on macrophage polarization (inhibited by activation of AMPK, Nrf2/HO-1 and AdipoR1 and AdipoR2 signaling pathway, upregulation of adiponectin, glucagon-like peptide-1 (GLP-1), interferon-gamma (IFN-γ), superoxide dismutases (SOD), catalase, and downregulation of NF-κ-B, leptin, IL-4, TNF-α, and IL-1). The browning of eWAT through the promotion of thermogenesis and activation of the melanocortin pathway also had a role in the reduction of obesity-related AHR. The findings of the study demonstrated that BCP reduced obesity-related AHR by inhibiting macrophage polarisation, activating AMPK, activating Nrf2/HO-1, increasing AdipoR1 and AdipoR2 expression, and decreasing NFB expression in the lungs of animals (Pathak et al., 2021).

## 4 Conclusion and prospects

The state of being obese is the reason behind many diseases where obesity is either primarily responsible or exaggerates the ongoing diseased condition. The pathogenesis of asthma in obese individuals is different from the normal weight individuals due to different genotypes and so responds poorly to the conventional asthma treatment. Although several mechanisms have been proposed, however, the exact mechanism behind the exaggeration of asthma in obese asthmatics is yet to unfurl. Studies found that for a better therapeutic outcome in obesity-associated asthma, pathogenesis related to obesity should also be targeted along with the asthma for a better outcome. Herbal medicines are known to possess a myriad of therapeutic approach together with low adverse effects. Although several bioactive phytoconstituents has demonstrated potential efficacy against obesity-associated asthma in pre-clinical models, however their number is less. In depth studies are warranted to decipher the pinpoint mechanisms to design effective therapeutic approach targeting the obesity-associated asthma. Similarly, it is high time to explore promising bioactive phytoconstituents having proven anti-obesity efficacy for amelioration of obesity-associated asthma in clinical setup for better understanding of their mechanism of actions.
